# Specific radiation damage is a lesser concern at room temperature

**DOI:** 10.1107/S205225251900616X

**Published:** 2019-06-12

**Authors:** Guillaume Gotthard, Sylvain Aumonier, Daniele De Sanctis, Gordon Leonard, David von Stetten, Antoine Royant

**Affiliations:** a European Synchrotron Radiation Facility, F-38043 Grenoble, France; b Université Grenoble Alpes, CNRS, CEA, IBS (Institut de Biologie Structurale), F-38000 Grenoble, France

**Keywords:** room-temperature macromolecular crystallography, cryocrystallography, specific radiation damage, time-resolved crystallography

## Abstract

Carrying out macromolecular crystallography (MX) experiments at cryogenic temperatures significantly slows the rate of global radiation damage, thus facilitating the solution of high-resolution crystal structures of macromolecules. However, cryo-MX experiments suffer from the early onset of so-called specific radiation damage that affects certain amino-acid residues and, in particular, the active sites of many proteins. Here, a series of MX experiments are described which suggest that specific and global radiation damage are much less decoupled at room temperature than they are at cryogenic temperatures. The results reported here demonstrate the interest in reviving the practice of collecting MX diffraction data at room temperature and allow structural biologists to favourably envisage the development of time-resolved MX experiments at synchrotron sources.

## Abstract 2

Both global and specific radiation damage have been investigated using three different proteins at cryogenic and room temperature. The large decoupling between global and specific radiation damage at cryogenic temperature appears to be practically abolished at room temperature, which has positive implications for time-resolved protein crystallography.

## 1. Introduction   

Radiation damage in macromolecular crystallography (MX) experiments is an unavoidable phenomenon. In the early days of MX this meant that it was necessary to compile a complete diffraction data set from partial data sets collected from several crystals, making structure solution a very lengthy and arduous process (Perutz *et al.*, 1960 ▸; Kendrew *et al.*, 1960 ▸; Blake *et al.*, 1965 ▸). The situation changed dramatically in the 1990s, when the advent of cryocooling (Hope, 1990 ▸; Garman & Schneider, 1997 ▸) greatly increased the absorbed dose that a crystal could tolerate before radiation damage destroyed its diffraction properties. This allowed the collection of complete, high-resolution diffraction data sets from a single crystal and paved the way for the explosion in the number and the types of macromolecular crystal structures that have been determined in the intervening quarter of a century. However, in the early 2000s a series of seminal papers (Ravelli & McSweeney, 2000 ▸; Burmeister, 2000 ▸; Weik *et al.*, 2000 ▸) showed that it was not only global radiation damage that was the enemy of the crystallographer. Indeed, two types of radiation damage could be defined: (i) global damage, which is mainly observed in reciprocal space and corresponds to a degradation of the diffraction properties of a crystal, and (ii) specific damage, which is mainly observed in real space and corresponds to the photoreduction of metal centres, the photoreduction of photoactive protein chromophores, the breakage of covalent bonds (for example disulfide bridges) and/or the loss of electron density for the side chains of some amino acids. These two phenomena are largely decoupled at cryogenic temperature. While acceptable absorbed dose limits for global radiation damage have been proposed to be in the range 20–30 MGy (Henderson, 1995 ▸; Owen *et al.*, 2006 ▸), specific radiation damage occurs at much lower absorbed doses [*e.g.* ∼7 kGy for the peroxo group in the active site of a reaction-intermediate state of urate oxidase (Bui *et al.*, 2014 ▸), ∼60 kGy for alteration of the chromophore in bacteriorhodopsin (Borshchevskiy *et al.*, 2014 ▸) and ∼1.6 MGy for the breakage of disulfide bonds in lysozyme (Carpentier *et al.*, 2010 ▸)]. This decoupling of global and specific radiation damage often means that extreme care should be taken when interpreting the results of structures determined by MX, and experimenters must be sure that the electron-density maps produced do not contain artefacts arising from specific radiation damage. Most often, this means that MX should be complemented with *in crystallo* optical spectroscopy (*ic*OS; von Stetten *et al.*, 2015 ▸) or other measurements which provide information on the changes in the chemical state of a macromolecule that can be induced by exposure to X-rays.

In addition to the potential artefacts induced by specific radiation damage, a second potential pitfall of cryocrystallography is the fact that the cryocooling process can artificially trap biologically inactive conformations of amino-acid side chains. This can also lead to the misinterpretation of enzyme mechanisms and of the roles of particular amino acids in specific biological processes (Fraser *et al.*, 2011 ▸). For this reason, room-temperature (RT) MX experiments are experiencing something of a renaissance. However, at RT the absorbed doses that induce global radiation damage can be two orders of magnitude lower than those at cryogenic temperatures (Nave & Garman, 2005 ▸; Southworth-Davies *et al.*, 2007 ▸), and a thorough characterization of the radiation sensitivity of the crystals under study should usually be carried out if a complete data set is to be collected from a single crystal at room temperature. The question of whether the dose rate has an effect on radiation sensitivity at room temperature has been extensively investigated, but has not received a clear answer for dose rates covering 50 Gy s^−1^ to 680 kGy s^−1^ (Southworth-Davies *et al.*, 2007 ▸; Rajendran *et al.*, 2011 ▸; Owen *et al.*, 2012 ▸; Warkentin *et al.*, 2012 ▸; Leal *et al.*, 2013 ▸). These investigations suggest, however, that the total absorbed dose has to be limited to a dose scale of hundreds of kilograys in order to record a complete data set, depending on the solvent content of the crystal (Leal *et al.*, 2013 ▸).

In the course of studying the chromophore-photoswitching behaviour of the fluorescent protein Cerulean (Gotthard *et al.*, 2017 ▸), we attempted to solve its room-temperature structure. Cerulean possesses a radiosensitive glutamate residue next to the chromophore, the side chain of which is severely affected by specific radiation damage (decarboxylation) during cryo-MX experiments. We thus expected this residue to be severely affected by specific radiation damage at RT. However, and to our surprise, the RT crystal structure that we obtained from a diffraction data set collected from a single crystal showed no sign of specific radiation damage. This prompted us to perform a more extensive study aimed at investigating specific radiation damage in room-temperature MX experiments. This topic was first considered by Southworth-Davies and coworkers when investigating the effects of various dose rates at room temperature (Southworth-Davies *et al.*, 2007 ▸) and then by Russi and coworkers in the course of comparing structural heterogeneity at room and cryogenic temperatures (Russi *et al.*, 2017 ▸). In both studies, it was observed that specific radiation damage was less obvious at room temperature than at cryogenic temperature. In this study, we recorded both X-ray diffraction data and, in order to obtain X-ray-independent estimates of specific radiation damage, complementary data using Raman or UV–Vis absorption spectroscopy. In addition to Cerulean, two other systems were studied: lysozyme, in order to examine the photoreduction of disulfide bonds at RT, and the photoadduct of the LOV2 domain of phototropin 2 from *Arabidopsis thaliana*, which upon light activation forms a covalent bond between the protein and its cofactor that is particularly sensitive to specific radiation damage at cryogenic temperatures. The results reported here suggest that specific and global radiation damage are much less decoupled at RT than they are at cryogenic temperature, thus confirming the interest in reviving the practice of collecting diffraction data at RT. They also allow structural biologists to favourably envisage the development of time-resolved MX experiments at synchrotron sources.

## 2. Methods   

### 2.1. Protein expression and purification   

Cerulean was overexpressed and purified using a previously described protocol (Lelimousin *et al.*, 2009 ▸; Gotthard *et al.*, 2017 ▸). Hen egg-white lysozyme (HEWL) was purchased from Roche Applied Science (catalogue No. 10837059001) and was dissolved in distilled water to a concentration of 40 mg ml^−1^. The gene coding for the LOV2 domain of phototropin 2 from *A. thaliana* (*At*Phot2LOV2) was synthesized (GeneCust, Ellange, Luxembourg) and inserted into a pBAD plasmid. The plasmid was transformed into an *Escherichia coli* BL21 strain and the cells were grown in 2 l ZYP-5052 autoinducible medium (Studier, 2005 ▸) at 37°C until the OD_600 nm_ reached 1.25. Protein expression was then induced with 0.2% l-arabinose for 14 h at 17°C. The cells were harvested by centrifugation (20 min at 4000*g*) and the pellets were resuspended in 25 ml of a lysis buffer consisting of 50 m*M* Tris pH 8.0, 300 m*M* NaCl, 10 m*M* imidazole, 0.25 mg ml^−1^ lysozyme, 400 µg ml^−1^ DNAse I, 20 m*M* MgSO_4_ and protease-inhibitor cocktail (cOmplete EDTA-free, Roche) and frozen at −80°C. The resuspended pellets were sonicated four times for 30 s at 35 W power (VC-750 ultrasonic processor, Bioblock Scientific) and the cell debris was harvested by centrifugation (40 min at 15 000*g* at 4°C). The protein was purified from the clarified lysate using a nickel-affinity column (HisTrap HP 5 ml, GE Healthcare, Little Chalfont, England) followed by size-exclusion chromatography (Superdex 75 10/300 GL, GE Healthcare). The purified *At*Phot2LOV2 was concentrated to 5 mg ml^−1^ and subjected to digestion with trypsin (1 h, ratio of 1:100) prior to crystallization.

### 2.2. Protein crystallization   

Cerulean was crystallized as described previously (Lelimousin *et al.*, 2009 ▸; Gotthard *et al.*, 2017 ▸) by the hanging-drop vapour-diffusion method (1:1 ratio in 2 µl drops) at 293 K using a protein concentration of 13 mg ml^−1^ in a condition consisting of 10–20% PEG 8000, 100 m*M* MgCl_2_, 100 m*M* HEPES pH 6.75–7.5. Needle-shaped crystals grew in five days and were used to seed subsequent optimized crystallization conditions (10–12% PEG 8000) by mixing the protein solution with the seed solution in a 1:10 or 1:100 ratio. Rod-shaped three-dimensional crystals then appeared after incubation for one week at 293 K. HEWL was crystallized using the sitting-drop vapour-diffusion method (1:1 ratio in 2 µl drops) in a crystallization condition consisting of 250–400 m*M* NaCl, 100 m*M* sodium acetate pH 4.8. Crystals belonging to the tetragonal space group *P*4_3_2_1_2 grew at 293 K within one week. *At*Phot2LOV2 was crystallized by the hanging-drop vapour-diffusion method (1:1 ratio in 2 µl drops) at 293 K using the protein at a concentration of 5 mg ml^−1^. Crystals appeared after two days in a condition consisting of 12–17% PEG 8000, 200 m*M* calcium acetate, 100 m*M* MES pH 6.0.

### 2.3. X-ray data collection   

The crystals were cryoprotected by transfer into a solution consisting of the reservoir solution diluted with 20%(*v*/*v*) glycerol (99.5% grade). X-ray data sets were recorded either at 100 K using an Oxford Cryostream 700 cryogenic system (Oxford Cryosystems, Oxford, England) or at room temperature using an HC1 humidity controller with a humidity level calculated from the composition of the mother liquor (Sanchez-Weatherby *et al.*, 2009 ▸). Crystal sizes and data-collection parameters are summarized in Table 1 ▸. X-ray data collections were carried out on beamlines ID29 (De Sanctis *et al.*, 2012 ▸), ID30B (McCarthy *et al.*, 2018 ▸) and ID30A-3 (Theveneau *et al.*, 2013 ▸) at the ESRF, which all have a Gaussian beam profile with an ellipsoid or circular shape. Data-collection statistics are reported in Tables 2 ▸, 3 ▸ and 4 ▸.

In order to perform the RT diffraction experiment on the unstable (in time, and potentially in dose) photoadduct of *At*Phot2LOV2, we mounted a single crystal on the goniometer of beamline ID30A-3 at the ESRF under the air flux of an HC1 humidity controller and then illuminated it with a 470 nm blue LED for 10 min in order to populate the crystal with the covalent intermediate. Upon starting data collection (Table 4 ▸) the LED was turned off (a process triggered by the trigger of the beamline’s EIGER 4M detector) and 1650° of oscillation data (corresponding to four complete data sets) were collected in a contiguous manner in a total of 10 s. Diffraction images corresponding to each of the four data sets were then processed as described below.

### 2.4. Data reduction and structure refinement   

The X-ray data sets were integrated, scaled and merged using the *XDS* program suite (Kabsch, 2010 ▸). Absorbed doses corresponding to the average dose of the exposed region were calculated using *RADDOSE*-3*D* (Zeldin *et al.*, 2013 ▸). The 1.15 Å resolution structure of Cerulean (PDB entry 2wso; Lelimousin *et al.*, 2009 ▸), the 1.2 Å resolution structure of lysozyme (PDB entry 5ebh; Zander *et al.*, 2016 ▸) and the 1.7 Å resolution structure of *At*Phot2LOV2 (PDB entry 4eep; Christie *et al.*, 2012 ▸) were used as starting models for molecular replacement or refinement. Models were manually rebuilt and refined using *Coot* (Emsley *et al.*, 2010 ▸) and *REFMAC*5 (Murshudov *et al.*, 2011 ▸), respectively. In order to quantify the extent of specific damage from the crystallo­graphic data, the default refinement protocol of *REFMAC*5 was modified by removing the restraint on *B*-factor continuity along covalent chains of atoms (Murshudov *et al.*, 2011 ▸). Data-collection and refinement statistics are shown in Tables 2 ▸, 3 ▸ and 4 ▸. Structural coordinates and structure factors were deposited in the PDB (http://www.pdb.org/) with the following accession codes: 6qq8, 6qq9, 6qqa, 6qqb, 6qqc, 6qqd, 6qqe, 6qqf, 6qqh, 6qqi, 6qqj, 6qqk and 6qsa. The decay of the *B*_0_/*B*_*n*_ ratio as a function of dose was modelled with a monoexponential decay behaviour *A* + *B*exp(−dose/τ), where τ is the life-dose or, when necessary, as a biexponential decay behaviour *A* + *B*exp(−dose/τ_1_) + *C*exp(−dose/τ_2_).

### 2.5. Online Raman spectroscopy   

Online Raman spectroscopy was performed on beamline ID29 as previously described using a setup specifically designed for the collection of X-ray and Raman data in an interleaved manner (von Stetten *et al.*, 2017 ▸). In brief, Raman spectra were recorded using an inVia Raman instrument (Renishaw, Wotton-under-Edge, Gloucestershire, England) equipped with a near-infrared (785 nm) 300 mW diode laser source. Raman spectra were measured from the X-ray-exposed region of a static lysozyme crystal with a composite acquisition time of 10 × 10 s for the 300–1800 cm^−1^ spectral window. Spectra were corrected for background using the *WiRE* software v.3.4 (Renishaw, Wotton-under-Edge, Gloucestershire, England). X-ray burn cycles were performed between Raman data sets by opening the shutter for increasing amounts of time, but no diffraction data were recorded. Absorbed doses were between 39 kGy and 1.21 MGy at 100 K and between 1.2 and 283 kGy at 293 K. The damage to disulfide bonds was evaluated by integrating the peak height of the 510 cm^−1^ band over the course of incremental dose absorption, the evolution of which with dose was modelled using a monoexponential decay behaviour *A* + *B*exp(−dose/τ).

### 2.6. *In crystallo* UV–visible absorption spectroscopy of LOV2   

Offline UV–Vis absorption spectra were recorded from an *At*Phot2LOV2 crystal at room temperature on the ESRF ID29S Cryobench microspectrophotometer (von Stetten *et al.*, 2015 ▸), using a high-sensitivity fixed-grating QE65Pro spectrophotometer with a back-thinned CCD detector (Ocean Optics, Dunedin, Florida, USA), a balanced deuterium–halogen DH2000-BAL light source (Ocean Optics, Dunedin, Florida, USA) and an HC1 humidity controller (Sanchez-Weatherby *et al.*, 2009 ▸), before and after illumination with a 470 nm LED at 28 W cm^−2^. Spectra were averaged from ten 400 ms acquisitions from ∼50 µm thick crystals.

Online UV–Vis absorption spectra were recorded on beamline ID30A-3 at the ESRF using a dedicated microspectrophotometer (McGeehan *et al.*, 2009 ▸) and were recorded using the same spectrophotometer and lamp from an *At*Phot2LOV2 crystal illuminated for 5 min with a 470 nm LED at 0.7 W cm^−2^ before flash-cooling at 100 K and subsequent X-ray irradiation. Spectra were recorded at 5 Hz (200 ms acquisition time) from a ∼50 µm thick crystal maintained in a fixed position. Each spectrum recorded under X-ray irradiation corresponded to an absorbed dose of 14 kGy.

## 3. Results   

### 3.1. Cerulean   

Fluorescent proteins of the green fluorescent protein family are β-barrel-shaped proteins that contain a fluorescent chromophore formed by the autocatalytic cyclization of three consecutive amino-acid residues and, as such, they provide convenient genetically encoded fluorescent reporters of localization or interaction *in cellulo* (Tsien, 1998 ▸). Tuning the properties of fluorescent proteins (colour, brightness, pH sensitivity *etc.*) by mutagenesis has been facilitated by the availability of crystal structures of fluorescent proteins, all of which were determined at cryogenic temperature. Nevertheless, RT crystal structures have also become available based on diffraction data collected either at synchrotrons (Kaucikas *et al.*, 2015 ▸) or at XFELs (Colletier *et al.*, 2016 ▸; Coquelle *et al.*, 2018 ▸; Hutchison *et al.*, 2017 ▸). The sensitivity of fluorescent proteins to specific X-ray-induced radiation damage at cryogenic temperature has been well documented, showing that the conserved glutamate residue close to the chromophore (Glu222 according to the GFP sequence) appears to be specifically affected at relatively low absorbed doses [*i.e.* ∼0.1, 0.8 and 0.1 MGy for IrisFP (Adam *et al.*, 2009 ▸), EGFP (Royant & Noirclerc-Savoye, 2011 ▸) and mNeonGreen (Clavel *et al.*, 2016 ▸), respectively]. Given the sensitivity of this residue in fluorescent proteins to specific radiation damage, the cyan fluorescent protein Cerulean (Rizzo *et al.*, 2004 ▸), which produces well diffracting crystals (Lelimousin *et al.*, 2009 ▸; Gotthard *et al.*, 2017 ▸), was chosen as the first target in the work described here.

#### 3.1.1. Radiation damage to Cerulean at cryogenic temperature (100 K)   

**Figure 1:**
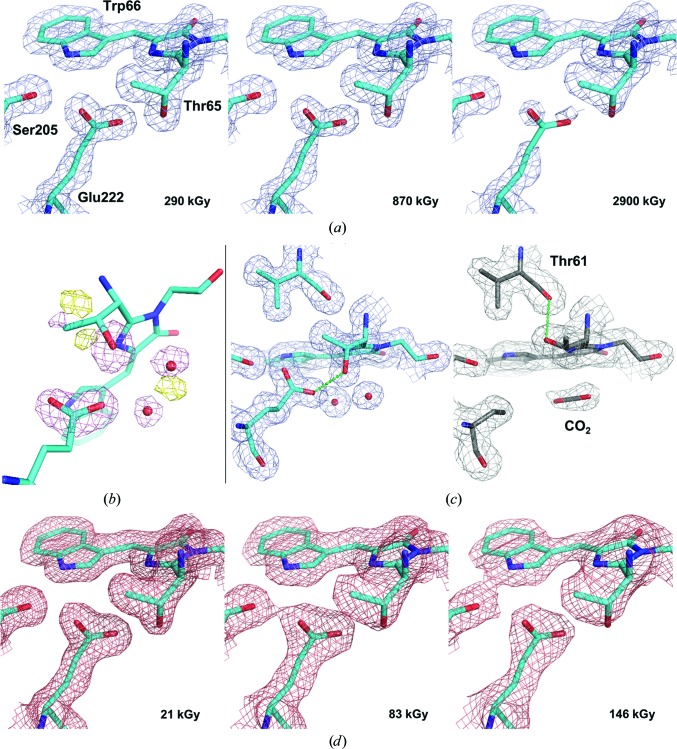
Evolution of the (2*mF*_obs_ − *DF*_calc_, α_calc_) electron-density map in data sets from a Cerulean crystal recorded at increasing absorbed doses at cryogenic and room temperature. (*a*) Series recorded at 100 K (maps contoured at a 1.5σ level). (*b*) [*F*_obs(20)_ − *F*_obs(1)_, α_calc(1)_] Fourier difference map calculated between the final and the initial 100 K data sets, highlighting the specific radiation damage to Glu222 and its structural consequences (maps contoured at a ±5.0σ level). (*c*) (2*mF*_obs_ − *DF*_calc_, α_calc_) electron-density map for the first (left) and the last (right) 100 K data sets, illustrating the decarboxylation process of Glu222 and the rotation of Thr65 (maps contoured at a 1.0σ level). (*d*) Series recorded at 293 K (maps contoured at a 1.5σ level).

In order to investigate specific damage in crystals of Cerulean at 100 K, 20 consecutive data sets corresponding to accumulated doses of between 290 kGy and 5.8 MGy were recorded from the same position of a single crystal (Table 2 ▸). As expected from studies of other fluorescent proteins (Adam *et al.*, 2009 ▸; Royant & Noirclerc-Savoye, 2011 ▸), Cerulean is sensitive to specific radiation damage at cryogenic temperatures and the progressive decarboxylation of Glu222 was observed in [2*mF*_obs(*i*)_ − *DF*_calc(*i*)_, α_calc(*i*)_] electron-density maps [Fig. 1 ▸(*a*)] and in the [*F*_obs(*i*)_ − *F*_obs(1)_, α_calc(1)_] Fourier difference map [Fig. 1 ▸(*b*)]. The progressive loss of this group results in rotation of the side chain of Thr65; the O^γ^ atom is originally engaged in a hydrogen bond to the carboxylate group of Glu222 [Fig. 1 ▸(*c*), left] and becomes engaged in a hydrogen bond to the carbonyl group of Thr61 [Fig. 1 ▸(*c*), right]. Two water molecules next to the Thr-Trp-Gly chromophore are also affected by the decarboxylation of Glu222, as shown by negative peaks in the [*F*_obs(20)_ − *F*_obs(1)_, α_calc(1)_] map [Fig. 1 ▸(*b*)]. Examination of the [2*mF*_obs(20)_ − *DF*_calc(20)_, α_calc(20)_] electron-density maps led us to replace these two water molecules by a linear carbon dioxide molecule, the obvious result of the photodecarboxylation process [Fig. 1 ▸(*d*)]. In order to compare the speed of global and specific radiation damage, we derived doses characteristic of each phenomenon from diffraction data and refined structure analysis.

**Figure 2:**
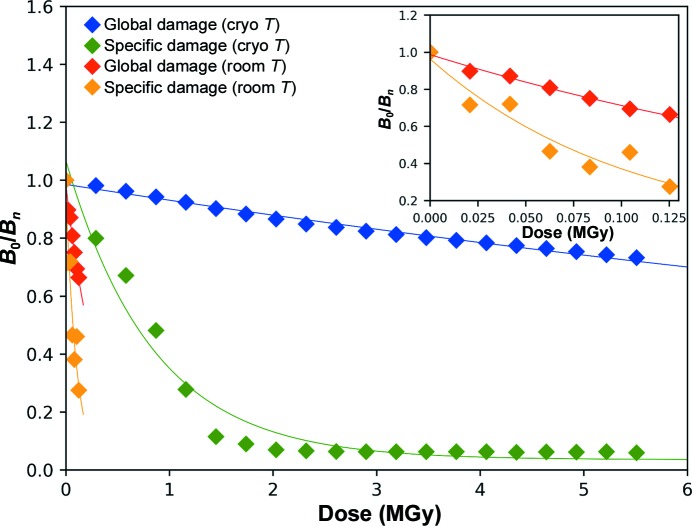
Evolution of *B* factors as a function of dose for the irradiation series of Cerulean crystals at cryogenic and room temperature. The evolution of the Wilson *B* factor (blue, 100 K; red, 293 K) represents the global radiation damage and the evolution of the atomic *B* factor of Glu222 C^δ^ (green, 100 K; orange, 293 K) illustrates the specific radiation damage to the carboxylate group of Glu222. An enlargement of the low-dose range is presented in the inset.

Diffraction resolution and *B*_Wilson_ are common markers of global radiation damage (Ravelli & McSweeney, 2000 ▸). Indeed, at 100 K the resolution of the data sets progressively decreased from *d*_min_ = 1.46 Å at an absorbed dose of 290 kGy to *d*_min_ = 1.82 Å at an absorbed dose of 5800 kGy, which amounts to a decrease in the diffraction power of the crystal. Owen and coworkers derived a half-dose *D*_1/2_ characteristic of global radiation damage by modelling a linear decay of the total diffracted intensities as a function of dose (Owen *et al.*, 2006 ▸), which we also derived for our crystals (Table 5 ▸). In addition to this diffracted intensity-based metric, we derived a second dose constant from the evolution of *B*_Wilson_(*n* = 1)/*B*_Wilson_(*n*) that we modelled as a monoexponential decay (blue trace in Fig. 2 ▸). We obtained a *B*_Wilson_-derived life-dose τ_Glob-CT_ of 17.6 MGy (where CT stands for ‘cryogenic temperature’), which we chose as a metric of global radiation damage to Cerulean at 100 K. Both values (Table 5 ▸) are consistent with the Henderson and Garman absorbed dose limits (20 and 30 MGy, respectively) for crystals of biological macromolecules (Henderson, 1995 ▸; Owen *et al.*, 2006 ▸).

To evaluate the characteristic dose at which specific radiation damage occurs at 100 K in crystals of Cerulean, we analysed the evolution of the atomic *B* factors of the progressively radiolysed carboxylate group of Glu222. C^δ^ is the only atom in the carboxylate group for which electron density eventually fully disappears [Fig. 1 ▸(*a*)]. All 20 structures were refined as if Glu222 had not been affected, with the smooth variation restraint on *B* factors of atoms implicated in consecutive covalent bonds (Murshudov *et al.*, 2011 ▸) specifically removed. The evolution of *B*_Glu222 Oδ_(*n* = 1)/*B*_Glu222 Oδ_(*n*) was then modelled as a monoexponential decay, resulting in a life-dose for specific radiation damage in crystals of Cerulean at 100 K (τ_Spec-CT_) of 843 kGy (green trace in Fig. 2 ▸).

The life-doses derived above indicate a strong decoupling between global and specific radiation damage in crystals of Cerulean at cryogenic temperature. In order to quantify this decoupling effect, we introduce the ratio between the global and the specific radiation-damage life-doses (Δ_G/S-CT_ = τ_Glob-CT_/τ_Spec-CT_), which we estimated at 20.8 for Cerulean at cryogenic temperature. Δ_G/S_ is thus the decoupling factor between global and specific radiation damage for a given protein at a given temperature. Life-doses and decoupling factors are summarized in Table 5 ▸.

#### 3.1.2. Radiation damage to Cerulean at room temperature (293 K)   

We performed a similar cumulative absorbed dose experiment on a Cerulean crystal maintained at room temperature during data collection [Table 2 ▸, Figs. 1 ▸(*d*) and 2 ▸]. We collected seven data sets with accumulated doses ranging between 21 and 146 kGy. As expected, there is a rapid drop-off in diffraction resolution (from *d*_min_ = 1.66 Å for the first data set to *d*_min_ = 2.45 Å for the last data set). The life-dose for global radiation damage at room temperature, τ_Glob-RT_, was calculated to be 308 kGy (red trace in Fig. 2 ▸), which was 57 times lower than that observed at 100 K and was consistent with previous estimations concerning global radiation-damage rates at room and cryogenic temperatures (a decrease by a factor of between 26 and 113; Nave & Garman, 2005 ▸; Southworth-Davies *et al.*, 2007 ▸). Intriguingly, however, (2*mF*_obs_ − *DF*_calc_, α_calc_) electron-density maps calculated from the successive cumulative dose data sets showed little, if any, sign of specific radiation damage [*i.e*. the decarboxylation of Glu222; Fig. 1 ▸(*d*)]. More surprisingly, calculation of successive [*F*_obs(*n*)_ − *F*_obs(1)_, α_calc(1)_] Fourier difference maps did not show a build-up of strong peaks with increasing values as is generally observed in the analysis of a cryogenic radiation-damage data-set series. This prevented us from comparing the rate of specific damage at both temperatures using a Fourier difference map-based approach (Carpentier *et al.*, 2010 ▸; Bury & Garman, 2018 ▸). Based on the evolution of the normalized atomic *B* factors of the atoms comprising the carboxyl group of Glu222, we derived the radiation-damage life-dose for this moiety at room temperature (τ_Spec-RT_; orange trace in Fig. 2 ▸) to be 105 kGy, a value relatively close to that for the global damage. This leads to a decoupling factor of Δ_G/S-RT_ = 2.9 for Cerulean at room temperature, which represents a sevenfold reduction of the radiation-damage decoupling between cryogenic and room temperatures. This observation is consistent with the lack of obvious visible evidence of specific radiation damage in crystals of Cerulean at room temperature. To investigate whether this observation is an isolated phenomenon, similar experiments were carried out on crystals of hen egg-white lysozyme, an archetypal radiation-damage test case.

### 3.2. Hen egg-white lysozyme   

Hen egg-white lysozyme (HEWL) is one of the first proteins in which specific radiation damage was identified (Weik *et al.*, 2000 ▸; Ravelli & McSweeney, 2000 ▸). The most prominent damage occurs to its four disulfide bonds, which lengthen owing to the formation of an anionic radical and eventually break (Weik *et al.*, 2002 ▸; Sutton *et al.*, 2013 ▸). Raman spectroscopy provides an X-ray-independent metric of damage to the disulfide bonds through monitoring of the Raman S—S stretching mode at 510 cm^−1^ (Carpentier *et al.*, 2010 ▸). The bi-exponential evolution of its decay revealed the presence of an X-ray-induced repair mechanism, in which the anionic radical can revert back to the oxidized state upon further irradiation.

#### 3.2.1. Radiation damage to hen egg-white lysozyme at cryogenic temperature (100 K)   

**Figure 3:**
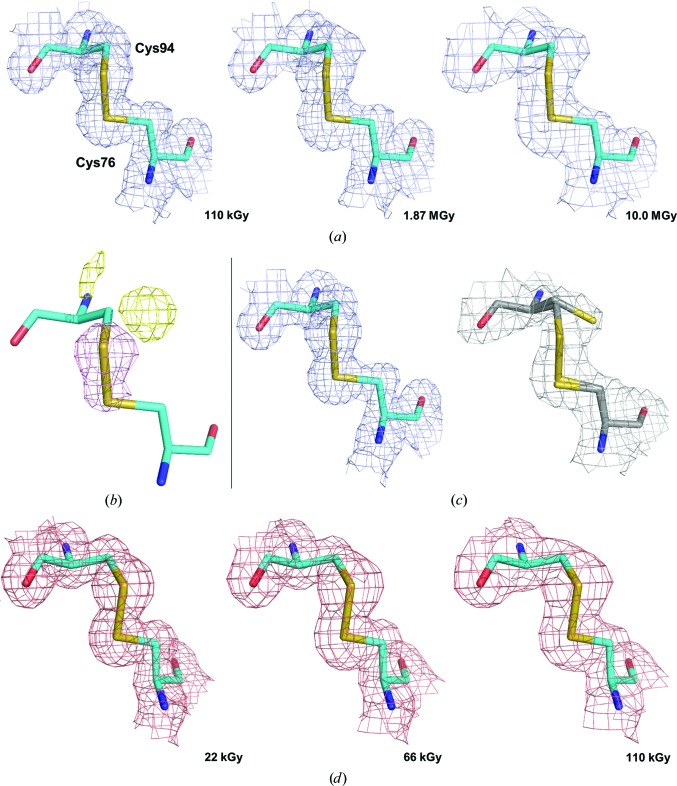
Specific radiation damage in crystals of HEWL. Evolution of the (2*mF*_obs_ − *DF*_calc_, α_calc_) electron-density map in data sets of lysozyme recorded with increasing doses at cryogenic and room temperature. (*a*) Series recorded at 100 K (maps contoured at a 0.6σ level). (*b*) [*F*_obs(6)_ − *F*_obs(1)_, α_calc(1)_] Fourier difference map calculated between the final and the initial data sets recorded at 100 K, highlighting the specific radiation damage to the disulfide bond Cys76–Cys94 (map contoured at a ±4.0σ level). (*c*) (2*mF*_obs_ − *DF*_calc_, α_calc_) electron-density map for the first (left) and the last (right) data sets recorded at 100 K, illustrating the breakage of the disulfide bond leading to the reorientation of the side chain of Cys94 (maps contoured at a 1.0σ level). (*d*) Series recorded at 293 K (maps contoured at a 1.0σ level).

**Figure 4:**
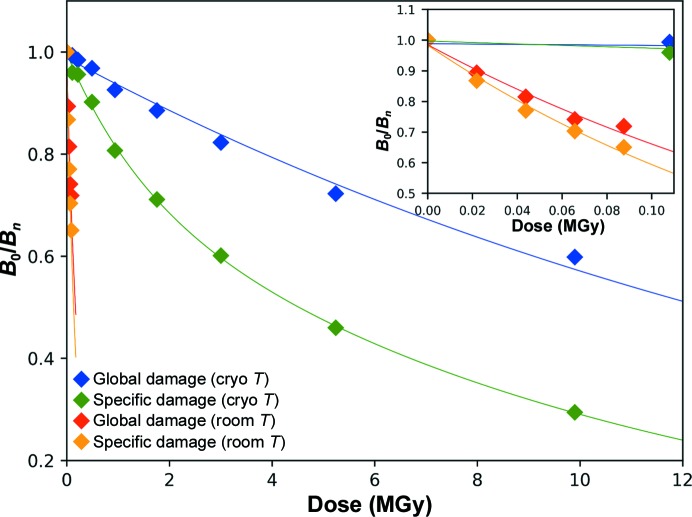
Evolution of *B* factors as a function of dose for the irradiation series of HEWL crystals at cryogenic and room temperature. The evolution of the Wilson *B* factor (blue, 100 K; red, 293 K) illustrates the global radiation damage and the evolution of the atomic *B* factor of Cys94 S^γ^ (green, 100 K; orange, 293 K) illustrates the specific radiation damage to the disulfide bond Cys76–Cys94. An enlargement of the low-dose range is presented in the inset.

Using the same approach as for Cerulean, nine consecutive data sets, corresponding to accumulated absorbed doses of between 110 kGy and 10.0 MGy, were collected at 100 K from the same position of a single crystal. Over the nine data sets collected, the resolution of the diffraction data decreased from *d*_min_ = 1.42 Å for the first data set to *d*_min_ = 1.92 Å for the last data set. Examination of the [2*mF*_obs(*i*)_ − *DF*_calc(*i*)_, α_calc(*i*)_] maps showed the expected progressive reduction of disulfide bonds, in particular for the Cys76–Cys94 disulfide bridge [Fig. 3 ▸(*a*)]. Based on the evolution of the Wilson *B* factors, τ_Glob-CT_ was estimated to be 18.2 MGy (blue trace in Fig. 4 ▸), consistent with the Henderson and Garman dose limits.

In order to calculate the life-dose of specific radiation damage at 100 K, we concentrated on the disulfide bond between Cys76 and Cys94, as its reduction leads to the largest observable structural change, which is the reorientation of the side chain of Cys94 upon breakage of the disulfide bond [Figs. 3 ▸(*b*) and 3 ▸(*c*)]. The evolution of the normalized atomic *B* factor of S^γ^ of Cys94 is, as expected (Carpentier *et al.*, 2010 ▸), best modelled by a bi-exponential decay, with a fast specific radiation-damage life-dose constant (τ_Spec-CT_) of 1.48 MGy and a slow one (τ′_Spec-CT_) of 10.5 MGy (green trace in Fig. 4 ▸). Thus, as observed for Cerulean, there is a clear decoupling between specific and global radiation damage in crystals of lysozyme at 100 K (Δ_G/S-CT_ = 12.4).

#### 3.2.2. Radiation damage to hen egg-white lysozyme at room temperature (293 K)   

To investigate radiation damage in crystals of HEWL at room temperature, five consecutive data sets corresponding to accumulated absorbed doses of between 22 and 110 kGy were collected from the same position of a single crystal (Table 3 ▸). Over the course of the five data sets collected, the resolution of the diffraction data decreased from *d*_min_ = 1.37 Å for the first data set to *d*_min_ = 1.89 Å for the last data set, and we derived a life-dose constant for global radiation damage at room temperature (τ_Glob-RT_) of 251 kGy (red trace in Fig. 4 ▸), which is approximately 73 times lower than that seen in our experiment at 100 K, and is again consistent with the 26–113 range of increase (Nave & Garman, 2005 ▸; Southworth-Davies *et al.*, 2007 ▸). However, the careful inspection of electron-density maps calculated for each of the five successive data sets showed no obvious sign of specific radiation damage to the Cys76–Cys94 disulfide bond [Fig. 3 ▸(*d*)], in accordance with previous observations (Southworth-Davies *et al.*, 2007 ▸; Russi *et al.*, 2017 ▸). Indeed, the evolution of the atomic *B* factor of S^γ^ of Cys94 can be best modelled with a life-dose τ_Spec-RT_ of 198 kGy (orange trace in Fig. 4 ▸), which gives a decoupling factor Δ_G/S-RT_ for lysozyme at room temperature of only 1.3, which constitutes an almost perfect concurrency between the two types of radiation damage.

#### 3.2.3. Spectroscopic investigations of specific radiation damage to hen egg-white lysozyme   

We were intrigued by the apparent absence of specific radiation damage in our room-temperature diffraction-based investigations of both Cerulean and HEWL. To further investigate this, a diffraction-independent method, Raman spectroscopy, was used to investigate the photoreduction of disulfide bonds in crystals of HEWL at room temperature by monitoring the Raman peak at 510 cm^−1^, which is assigned to disulfide bonds (Carpentier *et al.*, 2010 ▸).

**Figure 5:**
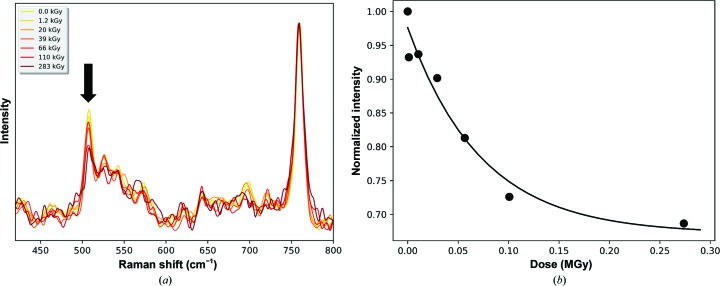
Specific X-ray damage in hen egg-white lysozyme probed by Raman spectroscopy. (*a*) Evolution of Raman spectra recorded with increasing X-ray doses from a lysozyme crystal at 293 K. The arrow indicates the only band whose intensity decreases with increasing dose. (*b*) Decay of the disulfide-bond stretching mode at 510 cm^−1^.

Online *in crystallo* nonresonant Raman spectra were recorded on beamline ID29 (von Stetten *et al.*, 2017 ▸) in an interleaved manner before and after X-ray burning cycles at room temperature (293 K). A decrease in the 510 cm^−1^ peak height is observed, demonstrating that specific radiation damage to disulfide bonds also occurs at 293 K [Figs. 5 ▸(*a*) and 5 ▸(*b*)]. Using the evolution of the 510 cm^−1^ peak height as a metric, the specific radiation-damage life-dose in crystals of HEWL at 293 K was calculated to be 89 kGy [Fig. 5 ▸(*b*)], which is of the same order as the life-dose derived from the diffraction-based experiments.

### 3.3. Photoadduct of the LOV2 domain from *A. thaliana* phototropin 2   

**Figure 6:**
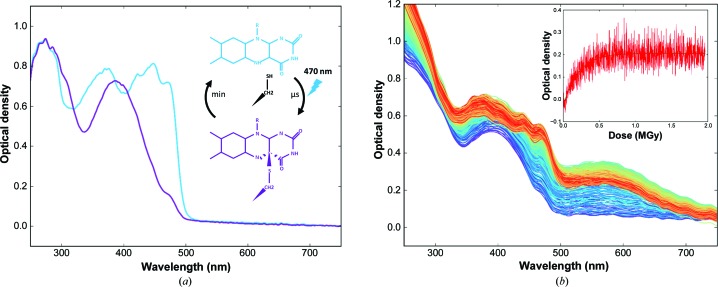
Spectroscopic characterization of the dark and light states of *At*Phot2LOV2. (*a*) Absorption spectra of the dark state (blue) and the blue-light-induced photoadduct (purple) recorded from crystals at room temperature. (*b*) Evolution of UV–Vis absorption spectra recorded with increasing X-ray doses from an *At*Phot2LOV2 crystal at 100 K (inset: dose-dependent evolution of the absorbance at 490 nm, illustrating the X-ray-induced relaxation to the dark state).

Light-, oxygen- or voltage-sensing (LOV) domains are protein modules that are found in higher plants, unicellular algae, fungi and bacteria that allow the sensing of environmental conditions (Christie *et al.*, 2015 ▸). In particular, they are found in the blue-light photoreceptor phototropin used by higher plants to mediate positive or negative growth towards or away from a light source. LOV domains contain a light-sensing chromophore, the flavin FMN, which forms a covalent adduct with a cysteine upon absorption of a blue light photon, while exhibiting a blue shift of its absorption maximum [Fig. 6 ▸(*a*)]. The crystal structures of various LOV-domain photoadducts have been determined both at room temperature under photostationary illumination (LOV2 domain of the chimeric phytochrome/phototropin phy3 from *Adiantum capillus-veneris*; Crosson & Moffat, 2002 ▸) and at cryogenic temperature (LOV1 domain of phototropin 1 from *Chlamydomonas reinhardtii*; Fedorov *et al.*, 2003 ▸). In the latter case, the radiosensitivity of the photoadduct required a composite data-set approach (*i.e.* the merging of partial data sets from several crystals). In the former, continuous illumination ensured a maximal population of the photoadduct in the crystal.

#### 3.3.1. High X-ray sensitivity of the *At*Phot2LOV2 photoadduct at 100 K   

**Figure 7:**
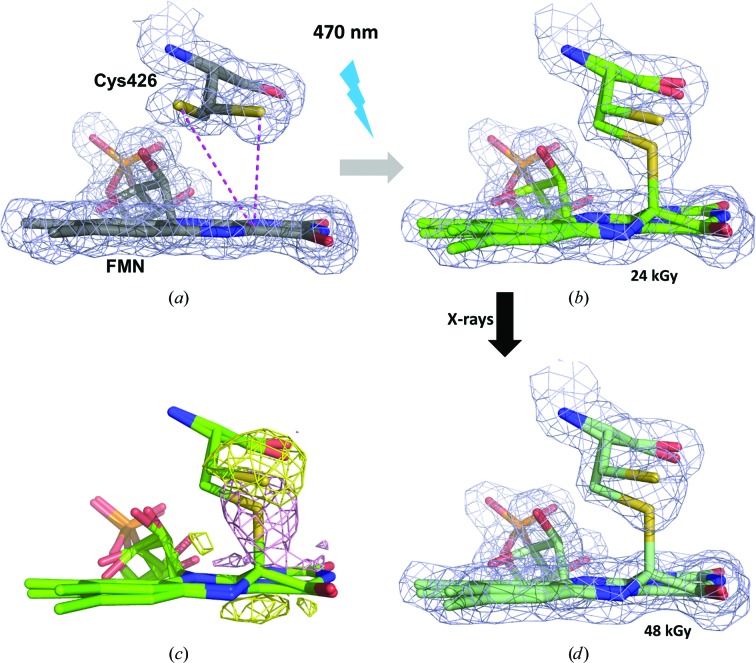
(2*mF*_obs_ − *DF*_calc_, α_calc_) electron-density map in data sets for the dark and light states of *At*Phot2LOV2 at 100 K. (*a*) Structure of the dark state at the position of the chromophore FMN (maps contoured at a 1.5σ level). (*b*) Structure of the light state recorded with an accumulated dose of 24 kGy (maps contoured at a 1.5σ level). (*c*) [*F*_obs(2)_ − *F*_obs(1)_, α_calc(1)_] Fourier difference map calculated between the second and the first data sets of the irradiation series on the *At*Phot2LOV2 light state, highlighting the very fast specific radiation damage to the Cys426–FMN covalent bond (map contoured at a ±4.0σ level). (*d*) Structure of the light state recorded with a total accumulated dose of 48 kGy (maps contoured at a 1.5σ level).

We chose the LOV2 domain of phototropin 2 from *A. thaliana* (*At*Phot2LOV2) and investigated the global and specific radiation-damage sensitivity of its photoadduct. We first determined the crystal structure of *At*Phot2LOV2 in its dark state at 1.38 Å resolution at cryogenic temperature. As can be seen in Fig. 7 ▸(*a*), the S^γ^ atom of the cysteine residue involved in photoadduct formation presents two alternate conformations: one in which the atom is at 3.6 Å from C^4α^ (65% occupancy) and one in which the S^γ^–C^4α^ distance is 4.3 Å (35% occupancy) [Fig. 7 ▸(*a*)].

**Figure 8:**
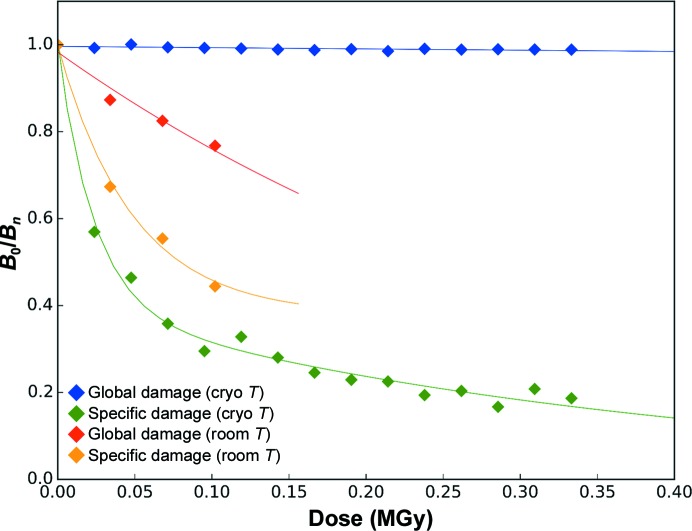
Evolution of *B* factors as a function of dose for the irradiation series of *At*Phot2LOV2 crystals at cryogenic and room temperature. The evolution of the Wilson *B* factor (blue, 100 K; red, 293 K) illustrates global radiation damage and the evolution of the atomic *B* factor of Cys426 S^γ^ (green, 100 K; orange, 293 K) illustrates the specific radiation damage to the covalent bond Cys426–FMN that occurs concurrently with the time-dependent relaxation of the photoadduct.

The photoadduct was generated in the crystal by illumination with a 470 nm LED just before flash-cooling. As for Cerulean and HEWL, we then collected 15 successive data sets from the same position of a single crystal, corresponding to accumulated absorbed doses of between 24 and 360 kGy, which constitutes a low-dose range (Table 4 ▸). Over the course of data-set collection, the diffraction limits hardly decreased from *d*_min_ = 1.70 Å for the first data set to *d*_min_ = 1.71 Å for the last data set, and we derived a life-dose τ_Glob-CT_ of 34.3 MGy (Fig. 8 ▸). As for the experiments described above for Cerulean and HEWL, this value is consistent with the Henderson and Garman limits.

Analysis of the crystal structure from the first data set (absorbed dose of 24 kGy) shows the presence of a mixture of the light-state structure with 50% occupancy (the presence of an S^γ^—C^4α^ covalent bond) and the dark-state structure with 50% occupancy [Fig. 7 ▸(*b*)], which results from the photo­stationary equilibrium obtained under continuous illumination before flash-cooling. The second data set (absorbed dose of 48 kGy) already shows significant photoreduction of the S^γ^—C^4α^ bond as shown by (2*mF*_obs_ − *DF*_calc_, α_calc_) electron-density maps and the Fourier difference map (*F*_obs2_ − *F*_obs1_, α_calc1_) calculated between data sets 1 and 2 [Figs. 7 ▸(*c*) and 7 ▸(*d*)]. The S^γ^ atom of Cys426 implicated in the covalent adduct in the light-state structure was chosen to derive the specific radiation-damage life-dose τ_Spec-CT_. The evolution of its *B* factor is best modelled by a bi-exponential decay, with a fast specific radiation-damage life-dose constant (τ_Spec-CT_) of 22 kGy and a slow one (τ′_Spec-CT_) of 387 kGy. The resulting decoupling factor Δ_G/S-CT_ of 1590 demonstrates that at 100 K this bond is two orders of magnitude more radiosensitive than the carboxylate group of Glu222 in Cerulean and the disulfide bond Cys94 in HEWL (Fig. 8 ▸).

In order to confirm these diffraction-based estimates, we used the complementary technique UV–Vis absorption microspectrophotometry (McGeehan *et al.*, 2009 ▸) on beamline ID30A-3 (MASSIF-3) at the ESRF (Theveneau *et al.*, 2013 ▸). Upon exposure to X-rays, the 390 nm peak characteristic of the light state changes into 450 and 470 nm peaks characteristic of the dark state [Fig. 6 ▸(*b*)]. This conversion can be interpreted as a result of X-ray-induced decay of the photoadduct species to the dark state [Fig. 7 ▸(*a*)]. We modelled the intensity increase of the 475 nm peak [inset in Fig. 6 ▸(*b*)] with the monoexponential behaviour *A* − *B*exp(−dose/τ), which gives a life-dose of 207 kGy for the phenomenon. This value compares well with the fast and slow life-doses of 22 and 387 kGy, respectively, derived from the diffraction data.

#### 3.3.2. The *At*Phot2LOV2 photo­adduct at room temperature   

**Figure 9:**
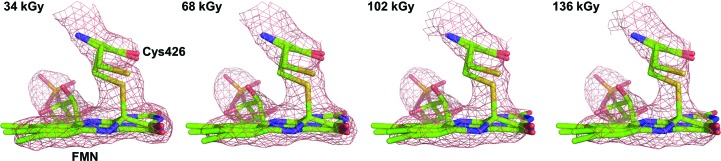
Evolution of the (2*mF*_obs_ − *DF*_calc_, α_calc_) electron-density map (maps contoured at a 2.0σ level) in data sets for *At*Phot2LOV2 recorded with increasing doses at room temperature.

While the *At*Phot2LOV2 photoadduct builds up on the microsecond time scale, its decay back to the dark state occurs on the second to minute time scale (Kasahara *et al.*, 2002 ▸), which opens up the possibility of determining its crystal structure at room temperature. We devised a strategy to record diffraction data as fast as possible in order to probe both global and specific radiation damage while minimizing the extent of intermediate-state relaxation. To this end, we used the EIGER 4M detector (Dectris, Switzerland) on beamline ID30A-3 (Theveneau *et al.*, 2013 ▸) to record diffraction data from a crystal of *At*Phot2LOV2 at RT immediately after blue-light irradiation. A continuous wedge of 1440° was recorded in 8.64 s, resulting in four successive 360° data sets, each of them for an absorbed dose of 34 kGy. Over the course of data collection, the resolution of the diffraction data as defined by the CC_1/2_ of the outer shell above 0.7 decreased from *d*_min_ = 2.40 Å to *d*_min_ = 2.78 Å. Representation of the four successive [2*mF*_obs(*i*)_ − *D* *F*_calc(*i*)_, α_calc(*i*)_] maps at a 1.5σ level does not show a major disappearance of the electron density corresponding to the S^γ^—C^4α^ covalent bond (Fig. 9 ▸). Based on the evolution of *B*_Wilson_ and of the atomic *B* factor of Cys426 S^γ^, we calculate RT life-doses τ_Glob-RT_ and τ_Spec-RT_ of 389 and 49 kGy, respectively (Fig. 8 ▸). The resulting decoupling factor Δ_G/S-RT_ of 8.0 constitutes a 200-fold reduction compared with the value at cryogenic temperature. Given that a significant part of the intermediate-state population has already relaxed even in 10 s, the ‘true’ value of Δ_G/S-RT_ has to be closer to those observed for Cerulean (Δ_G/S-RT_ = 2.9) and lysozyme (Δ_G/S-RT_ = 1.3) (Table 5 ▸). This means that in all three of our cases the decoupling factor between global and radiation damage is less than 10 and is probably close to 1, posing the question of the severity of specific radiation damage at room temperature.

## 4. Discussion   

Our initial inability to detect traces of specific radiation damage in room-temperature diffraction data sets from single crystals of the fluorescent protein Cerulean prompted us to define a diffraction-based metric that would allow an easy comparison of the appearance of specific damage in different systems at various temperatures. To this end, the decoupling factor Δ_G/S-T_, which uses the onset of global damage for normalization at a given temperature *T*, was introduced. This ratio was calculated for both Cerulean and the well studied protein lysozyme at cryogenic and room temperature. At cryogenic temperature, the decoupling factors of 12 and 21, respectively, illustrate that the partial decarboxylation of a key glutamate residue in Cerulean and the partial reduction of a disulfide bond in lysozyme occur at moderate doses, well before the effects of global damage are apparent. At room temperature, however, the decoupling factors decrease to much lower values (1 and 3, respectively), suggesting a ‘recoupling’ of specific and global damage. In order to investigate whether this phenomenon also holds true for more radiosensitive systems, the same analysis was performed on the photoadduct of a phototropin LOV2 domain. Here, a huge decoupling factor of 1590 at cryogenic temperature reduces to a decoupling factor of only 8 at room temperature. In other words, and in contrast to the situation at cryogenic temperature, specific and global radiation damage evolve on similar dose scales at room temperature for these three systems, all of which involve covalent-bond breakage. Specific damage thus may appear to be as random as global damage, which would for instance explain why it does not show in Fourier difference maps.

Further studies, including simulations, are required to understand the mechanisms that cause the difference in behaviour at cryogenic versus room temperature. A potential reason may reside in the free-energy landscape offered at the respective temperatures to the solvated electrons and the free radicals that are generated by the interaction of X-rays with bulk-solvent molecules. Escape lanes for solvated electrons and free radicals terminate either on random groups, resulting in global damage, or on a few groups that are particularly reactive towards electrons and radicals, resulting in specific damage. The key difference between free radicals and solvated electrons is that the former are trapped, and therefore mostly inactive at cryogenic temperature, while the latter can still diffuse (Kmetko *et al.*, 2011 ▸) and be funnelled towards electron-avid groups within the protein. At room temperature, however, all free radicals created in the bulk-solvent region can diffuse and impair crystalline order through various mechanisms such as the perturbation of crystalline contacts through direct damage to the protein or the generation of gas molecules (Garman, 2010 ▸). The recoupling of specific and global damage at room temperature suggests that the specificity of certain X-ray-induced damage to proteins may only arise at cryogenic temperature.

These results are of considerable interest for genuinely time-resolved protein crystallography, which is performed at room temperature (in contrast to methods relying on the cryo-trapping of intermediate states) and which requires the determination of the structures of particularly X-ray-sensitive intermediate states. Indeed, the structure determination of reaction-intermediate states trapped at cryogenic temperature has often required great care in minimizing, or controlling, the deposited dose (Berglund *et al.*, 2002 ▸; Matsui *et al.*, 2002 ▸; Adam *et al.*, 2004 ▸; Bui *et al.*, 2014 ▸). The ‘recoupling’ of specific and global radiation damage at room temperature will make specific damage much less of a problem, provided that a full data set can be recorded from a single crystal, which can be tested by monitoring parameters indicative of global damage, an option that is now readily accessible on synchrotron beamlines using software such as *Dozor* (Zander *et al.*, 2015 ▸). An added advantage is that achieving the determination of a room-temperature structure of a given protein at a sufficiently high resolution will allow, by comparison, the identification of potential pitfalls in mechanism interpretation owing to specific radiation damage occurring in a cryogenic structure of the same protein. In summary, the development of easy-to-use humidity-controlled crystal environments and of fast and noise-free X-ray detectors is triggering a rebirth of room-temperature crystallography, which should be favoured in projects where obtaining a structure close to the structure in the physiological state is more important than reaching the highest resolution possible.

In conclusion, if one wishes to determine the structure of a protein whose active site (or another part) is particularly sensitive to X-rays, one can either work at cryogenic temperature and perform a thorough radiation-damage study by recording positive and negative control data sets aimed at closely monitoring the geometry of the active site (with the aim of deriving the maximum dose below which one should accumulate a complete data set) or work at room temperature and only focus on adjusting the X-ray flux to obtain a complete data set at the price of a reduced diffraction resolution.

## Supplementary Material

PDB reference: Cerulean, cryogenic temperature structure, accumulated dose 290 kGy, 6qq8

PDB reference: cryogenic temperature structure, accumulated dose 5.8 MGy, 6qq9

PDB reference: room-temperature structure, accumulated dose 21 kGy, 6qqa

PDB reference: room-temperature structure, accumulated dose 147 kGy, 6qqb

PDB reference: hen egg-white lysozyme, cryogenic temperature structure, accumulated dose 110 kGy, 6qqc

PDB reference: cryogenic temperature structure, accumulated dose 10 MGy, 6qqd

PDB reference: room-temperature structure, accumulated dose 20 kGy, 6qqe

PDB reference: room-temperature structure, accumulated dose 100 kGy, 6qqf

PDB reference: *At*Phot2LOV2, ground state, cryogenic temperature structure, accumulated dose 2.68 MGy, 6qqh

PDB reference: blue light-irradiated, cryogenic temperature structure, accumulated dose 24 kGy, 6qqi

PDB reference: ground state, room-temperature structure, accumulated dose 384 kGy, 6qqj

PDB reference: blue light-irradiated, room-temperature structure, accumulated dose 34 kGy, 6qqk

PDB reference: blue light-irradiated, cryogenic temperature structure, accumulated dose 48 kGy, 6qsa

## Figures and Tables

**Table 1:** Data-collection parameters

		Crystal dimensions (µm)										
Protein	Temperature (K)	*x*	*y*	*z*	Relative humidity level (%)	*a*, *b*, *c* (Å)	Beamline	Beam size (µm)	Energy (keV)	Initial flux (photons s^−1^)	Wedge (°)	Exposure time (s)
Cerulean	100	92	194	106	—	51.1, 62.7, 70.3	ID29	30 × 40	12.7	2.3 × 10^11^	90–290	40
	293	170	230	170	99	51.9, 63.0, 71.3	ID29	30 × 40	12.7	2.7 × 10^10^	90–290	40
Lysozyme	100	75	93	75	—	77.6, 77.6, 37.1	ID30B	40 × 40	12.7	3.4 × 10^10^	12–102	18
	293	218	379	202	98	79.2, 79.2, 38.1	ID29	30 × 40	11.5	4.1 × 10^10^	0–100	20
*At*Phot2LOV2 (dark state)	100	50	50	50	—	40.1, 40.1, 131.5	ID30A-3	15 × 15	12.8	3.5 × 10^11^	0–107	43
	293	50	50	50	98	40.9, 40.9, 132.7	ID30A-3	15 × 15	12.8	1.4 × 10^11^	0–120	14
*At*Phot2LOV2 (light state)	100	75	76	82	—	40.3, 40.3, 131.3	ID29	30 × 40	12.7	1.1 × 10^10^	87–177	36
	293	70	70	70	98	41.5, 41.5, 133.5	ID30A-3	15 × 15	12.8	2.0 × 10^11^	0–360	2.2

**Table:** Values in parentheses are for the outer shell.

	D1-100K	D2-100K	D3-100K	D4-100K	D5-100K	D6-100K	D7-100K	D8-100K	D9-100K	D10-100K
Data collection										
Temperature (K)	100									
Accumulated dose (MGy)	0.29	0.58	0.87	1.16	1.45	1.74	2.03	2.32	2.61	2.9
ESRF beamline	ID29									
Wavelength (Å)	0.976									
Space group	*P*2_1_2_1_2_1_									
*a*, *b*, *c* (Å)	51.11, 62.72, 70.34	51.13, 62.74, 70.37	51.15, 62.67, 70.39	51.16, 62.78, 70.41	51.18, 62.80, 70.42	51.20, 62.82, 70.43	51.22, 62.84, 70.45	51.21, 62.82, 70.41	51.25, 62.87, 70.47	51.27, 62.88, 70.48
Resolution range† (Å)	46.82–1.46 (1.50–1.46)	46.83–1.47 (1.51–1.47)	46.84–1.48 (1.52–1.48)	46.86–1.50 (1.54–1.50)	46.87–1.52 (1.56–1.52)	46.88–1.55 (1.59–1.55)	46.89–1.57 (1.61–1.57)	46.90–1.60 (1.64–1.60)	46.91–1.63 (1.67–1.63)	46.93–1.66 (1.70–1.66)
Wilson *B* factor (Å^2^)	28.345	28.876	29.474	30.075	30.696	31.433	32.101	32.736	33.41	33.874
Unique reflections	39910 (2939)	39156 (2843)	38421 (2778)	36921 (2648)	35579 (2618)	3393 (2445)	32406 (2390)	30647 (2218)	29061 (2114)	27543 (2002)
Multiplicity	7.1 (7.3)	7.0 (7.3)	7.0 (7.3)	7.0 (7.2)	7.0 (7.2)	7.0 (7.1)	7.0 (7.0)	7.0 (6.7)	7.0 (6.5)	7.0 (7.2)
Completeness (%)	99.9 (99.9)	99.9 (99.8)	99.9 (99.9)	99.8 (99.9)	99.8 (99.9)	99.8 (99.8)	99.8 (99.7)	99.8 (99.6)	99.8 (99.3)	99.9 (100.0)
Mean *I*/σ(*I*)	24.07 (1.56)	24.42 (1.51)	24.93 (1.45)	25.52 (1.58)	26.21 (1.56)	27.72 (1.66)	28.31 (1.73)	29.55 (1.83)	30.32 (1.82)	31.42 (2.04)
*R*_meas_ ‡	0.042 (1.387)	0.041 (1.437)	0.040 (1.478)	0.040 (1.379)	0.039 (1.370)	0.038 (1.272)	0.037 (1.216)	0.036 (1.130)	0.036 (1.144)	0.036 (1.103)
CC_1/2_	0.999 (0.724)	1.0 (0.697)	1.0 (0.709)	1.0 (0.750)	1.0 (0.705)	1.0 (0.703)	1.0 (0.712)	1.0 (0.728)	1.0 (0.709)	1.0 (0.717)
Refinement statistics										
Resolution (Å)	46.81–1.46 (1.50–1.46)									
*R*_work_	0.167 (0.42)									
*R*_free_	0.197 (0.38)									
No. of atoms	2161									
Average atomic *B* factor (Å^2^)	23.47									
R.m.s. deviations										
Bond lengths (Å)	0.012									
Bond angles (°)	1.63									
PDB code	6qq8									

**Table:** 

	D11-100K	D12-100K	D13-100K	D14-100K	D15-100K	D16-100K	D17-100K	D18-100K	D19-100K	D20-100K
Data collection										
Temperature (K)	100									
Accumulated dose (MGy)	3.19	3.48	3.77	4.06	4.35	4.64	4.93	5.22	5.51	5.80
ESRF beamline	ID29									
Wavelength (Å)	0.976									
Space group	*P*2_1_2_1_2_1_									
*a*, *b*, *c* (Å)	51.28, 62.90, 70.48	51.30, 62.91, 70.49	51.32, 62.93, 70.50	51.33, 62.94, 70.51	51.35, 62.96, 70.51	51.36, 62.97, 70.52	51.33, 62.94, 70.51	51.39, 62.99, 70.53	51.40, 63.00, 70.53	51.41, 63.00, 70.53
Resolution range† (Å)	46.93–1.68 (1.72–1.68)	46.94–1.70 (1.74–1.70)	46.95–1.72 (1.76–1.72)	46.95–1.74 (1.79–1.74)	46.97–1.76 (1.81–1.76)	46.97–1.78 (1.83–1.78)	46.96–1.79 (1.84–1.79)	46.99–1.80 (1.85–1.80)	46.99–1.81 (1.86–1.81)	46.99–1.82 (1.87–1.82)
Wilson *B* factor (Å^2^)	34.4	34.9	35.4	35.8	36.2	36.6	37.1	37.6	38.2	38.7
Unique reflections	26603 (1935)	25703 (1848)	24856 (1814)	24055 (1760)	23260 (1697)	22518 (1644)	22113 (1613)	21797 (1588)	21457 (1562)	21133 (1544)
Multiplicity	7.0 (7.4)	6.9 (7.4)	6.9 (7.4)	6.9 (7.4)	6.8 (7.4)	6.8 (7.4)	6.8 (7.4)	6.8 (7.4)	6.8 (7.3)	6.8 (7.3)
Completeness (%)	99.9 (100.0)	99.9 (99.9)	99.9 (99.9)	99.9 (100.0)	99.8 (99.9)	99.9 (100.0)	99.8 (100.0)	99.8 (99.9)	99.9 (100.0)	99.9 (100.00)
Mean *I*/σ(*I*)	30.87 (2.09)	31.11 (2.06)	30.77 (2.20)	31.25 (2.19)	31.10 (2.24)	31.16 (2.27)	30.41 (2.17)	29.48 (2.12)	28.85 (2.08)	28.23 (2.05)
*R*_meas_ ‡	0.037 (1.079)	0.037 (1.078)	0.037 (1.068)	0.037 (1.060)	0.038 (1.030)	0.038 (1.018)	0.039 (1.066)	0.040 (1.073)	0.041 (1.072)	0.042 (1.088)
CC_1/2_	1.0 (0.721)	1.0 (0.760)	1.0 (0.758)	1.0 (0.745)	1.0 (0.719)	1.0 (0.775)	1.0 (0.744)	1.0 (0.767)	1.0 (0.774)	1.0 (0.726)
Refinement statistics										
Resolution (Å)										46.99–1.82 (1.87–1.82)
*R*_work_										0.154 (0.281)
*R*_free_										0.195 (0.345)
No. of atoms										2220
Average atomic *B* factor (Å^2^)										33.9
R.m.s. deviations										
Bond lengths (Å)										0.009
Bond angles (°)										1.38
PDB code										6qq9

**Table:** 

	D1-293K	D2-293K	D3-293K	D4-293K	D5-293K	D6-293K	D7-293K
Data collection							
Temperature (K)	293						
Accumulated dose (MGy)	0.021	0.042	0.063	0.084	0.105	0.125	0.146
ESRF beamline	ID29						
Wavelength (Å)	0.976						
Space group	*P*2_1_2_1_2_1_						
*a*, *b*, *c* (Å)	51.93, 63.04, 71.32	51.99, 62.91, 71.45	52.08, 62.94, 71.58	52.11, 62.91, 71.64	52.12, 62.87, 71.68	52.09, 62.77, 71.63	52.04, 62.67, 71.56
Resolution range† (Å)	47.24–1.66 (1.70–1.66)	47.22–1.81 (1.86–1.81)	47.27–1.95 (2.00–1.95)	47.28–2.07 (2.12–2.07)	47.27–2.18 (2.24–2.18)	47.21–2.32 (2.38–2.32)	47.15–2.45 (2.51–2.45)
Wilson *B* factor (Å^2^)	33.1	36.9	38.1	41.0	44.1	47.7	49.9
Unique reflections	28306 (2062)	21964 (1592)	17737 (1296)	14881 (1060)	12788 (912)	10628 (773)	9048 (670)
Multiplicity	7.3 (7.4)	7.3 (7.4)	7.1 (7.1)	7.1 (6.8)	7.0 (7.7)	6.8 (7.6)	6.6 (7.4)
Completeness (%)	100.0 (100.0)	100.0 (99.9)	99.9 (100.0)	99.9 (99.6)	100.0 (100.0)	99.9 (100.0)	99.9 (100.00)
Mean *I*/σ(*I*)	15.43 (2.09)	21.87 (2.13)	12.01 (2.06)	10.65 (2.06)	9.71 (2.03)	9.65 (2.09)	8.90 (2.08)
*R*_meas_ ‡	0.07 (1.053)	0.06 (1.190)	0.132 (1.534)	0.155 (1.501)	0.168 (1.610)	0.174 (1.562)	0.185 (1.425)
CC_1/2_	0.998 (0.753)	1.00 (0.788)	0.998 (0.731)	0.997 (0.641)	0.997 (0.774)	0.997 (0.815)	0.997 (0.749)
Refinement statistics							
Resolution (Å)	47.23–1.66 (1.70–1.66)						47.15–2.45 (2.51–2.45)
*R*_work_	0.144 (0.254)						0.256 (0.386)
*R*_free_	0.171 (0.285)						0.281 (0.417)
No. of atoms	2161						2161
Average atomic *B* factor (Å^2^)	31.3						50.3
R.m.s. deviations							
Bond lengths (Å)	0.01						0.006
Bond angles (°)	1.65						1.31
PDB entry	6qqa						6qqb

† The resolution cutoff is based on CC_1/2_.

‡ *R*_meas_ = *R*_merge_ × [*N*/(*N* − 1)]^1/2^, where *N* is the data multiplicity.

**Table:** Values in parentheses are for the outer shell.

	D1-100K	D2-100K	D3-100K	D4-100K	D5-100K	D6-100K	D7-100K	D8-100K	D9-100K
Data collection									
Temperature (K)	100								
Accumulated dose (MGy)	0.11	0.22	0.33	0.60	1.05	1.87	3.12	5.35	10.01
ESRF beamline	ID29								
Wavelength (Å)	0.976								
Space group	*P*4_3_2_1_2								
*a*, *b*, *c* (Å)	77.56, 77.56, 37.13	77.56, 77.56, 37.14	77.58, 77.58, 37.15	77.60, 77.60, 37.16	77.64 77.64, 37.18	77.69, 77.69, 37.21	77.77, 77.77, 37.25	77.87, 77.87, 37.31	77.96, 77.96, 37.38
Resolution range† (Å)	33.49–1.42 (1.46–1.42)	33.50–1.43 (1.47–1.43)	33.51–1.43 (1.47–1.43)	33.52–1.44 (1.48–1.44)	33.54–1.49 (1.53–1.49)	33.56–1.52 (1.56–1.52)	33.60–1.58 (1.62–1.58)	33.65–1.72 (1.76–1.72)	33.71–1.92 (1.97–1.92)
Wilson *B* factor (Å^2^)	23.1	23.3	23.5	23.9	25.0	26.7	28.1	32.0	38.7
Unique reflections	20446 (1472)	20032 (1407)	20027 (1419)	19659 (1405)	17827 (1291)	16874 (1242)	15081 (1124)	11760 (893)	8490 (640)
Multiplicity	6.5 (6.8)	6.5 (6.8)	6.5 (6.8)	6.5 (6.9)	6.5 (6.7)	6.5 (6.6)	6.5 (6.1)	6.5 (6.4)	6.4 (6.6)
Completeness (%)	93.1 (92.5)	93.2 (91.8)	93.1 (92.0)	93.1 (92.1)	93.2 (94.2)	93.3 (94.1)	93.1 (94.6)	92.6 (96.3)	91.8 (95.7)
Mean *I*/σ(*I*)	17.49 (1.99)	17.28 (2.03)	17.51 (1.96)	17.07 (1.96)	17.87 (2.12)	17.48 (1.96)	17.15 (1.86)	16.93 (1.98)	15.34 (1.88)
*R*_meas_ ‡	0.059 (0.836)	0.060 (0.816)	0.059 (0.842)	0.060 (0.837)	0.058 (0.791)	0.059 (0.835)	0.062 (0.909)	0.065 (0.878)	0.078 (1.015)
CC_1/2_	1.000 (0.703)	0.999 (0.726)	1.000 (0.710)	1.000 (0.726)	1.000 (0.724)	1.000 (0.713)	0.999 (0.718)	0.999 (0.711)	0.999 (0.706)
Refinement statistics									
Resolution (Å)	33.49–1.42 (1.46–1.42)								33.71–1.92 (19.7–1.92)
*R*_work_	0.169 (0.262)								0.156 (0.258)
*R*_free_	0.203 (0.288)								0.216 (0.267)
No. of atoms	1212								1217
Average atomic *B* factor (Å^2^)	22.3								40.9
R.m.s. deviations									
Bond lengths (Å)	0.011								0.009
Bond angles (°)	1.78								1.572
PDB code	6qqc								6qqd

**Table:** 

	D1-293K	D2-293K	D3-293K	D4-293K	D5-293K
Data collection					
Temperature (K)	293				
Accumulated dose (MGy)	0.020	0.040	0.060	0.080	0.100
ESRF beamline	ID29				
Wavelength (Å)	1.07				
Space group	*P*4_3_2_1_2				
*a*, *b*, *c* (Å)	79.24, 79.24, 38.06	79.15, 79.15, 38.10	79.07, 79.07, 38.12	78.88, 78.88, 38.09	79.01, 79.01, 38.18
Resolution range† (Å)	39.62–1.37 (1.41–1.37)	39.58–1.49 (1.53–1.49)	39.54–1.59 (1.63–1.59)	39.44–1.80 (1.85–1.80)	39.51–1.95 (2.00–1.95)
Wilson *B* factor (Å^2^)	25.0	28.0	30.7	33.7	34.7
Unique reflections	25762 (1828)	20176 (1450)	16682 (1201)	11545 (813)	9218 (665)
Multiplicity	7.0 (7.0)	7.0 (7.3)	7.0 (7.2)	6.6 (7.1)	6.7 (7.2)
Completeness (%)	98.9 (96.9)	99.2 (97.8)	99.4 (99.8)	99.3 (98.1)	99.6 (99.7)
Mean *I*/σ(*I*)	17.10 (2.24)	16.75 (2.43)	15.84 (2.31)	13.62 (2.54)	10.45 (2.73)
*R*_meas_ ‡	0.055 (0.810)	0.059 (0.816)	0.066 (0.863)	0.108 (1.122)	0.152 (1.138)
CC_1/2_	0.999 (0.70)	0.999 (0.725)	0.999 (0.715)	0.998 (0.725)	0.997 (0.708)
Refinement statistics					
Resolution (Å)	39.61–1.37 (1.41–1.37)				39.51–1.95 (2.00–1.95)
*R*_work_	0.165 (0.271)				0.167 (0.223)
*R*_free_	0.195 (0.255)				0.217 (0.230)
No. of atoms	1221				1143
Average atomic *B* factor (Å^2^)	20.4				28.0
R.m.s. deviations					
Bond lengths (Å)	0.05				0.009
Bond angles (°)	1.10				1.27
PDB code	6qqe				6qqf

† The resolution cutoff is based on CC_1/2_.

‡ *R*_meas_ = *R*_merge_ × [*N*/(*N* − 1)]^1/2^, where *N* is the data multiplicity.

**Table:** Values in parentheses are for the outer shell.

	Dark-100K	Light-100K D1	Light-100K D2	Light-100K D3	Light-100K D4	Light-100K D5	Light-100K D6	Light-100K D7
Data collection								
Temperature (K)	100				100			
Accumulated dose (MGy)	2.68	0.024	0.048	0.071	0.095	0.119	0.143	0.167
ESRF beamline	ID30A-3				ID29			
Wavelength (Å)	0.968				0.976			
Space group	*P*4_3_2_1_2							
*a*, *b*, *c* (Å)	40.15, 40.15, 131.57	40.32, 40.32, 131.28	40.33, 40.33, 131.28	40.32, 40.32, 131.28	40.38, 40.38, 131.40	40.38, 40.38, 131.41	40.38, 40.38, 131.41	40.38, 40.38, 131.41
Resolution range† (Å)	38.40–1.38 (1.43–1.38)	38.55–1.70 (1.76–1.70)	38.55–1.70 (1.76–1.70)	38.55–1.70 (1.76–1.70)	38.60–1.71 (1.77–1.71)	38.60–1.71 (1.77–1.71)	38.61–1.71 (1.77–1.71)	38.61–1.71 (1.77–1.71)
Wilson *B* factor (Å^2^)	18.8	29.1	29.1	29.1	29.3	29.4	29.4	29.5
Unique reflections	23065 (2265)	12655 (1202)	12659 (1199)	12667 (1207)	12393 (1204)	12383 (1201)	12396 (1200)	12382 (1197)
Multiplicity	7.18 (6.32)	6.24 (6.47)	6.24 (6.45)	6.24 (6.47)	6.29 (6.45)	6.29 (6.43)	6.28 (6.43)	6.29 (6.41)
Completeness (%)	99.8 (99.8)	99.7 (99.0)	99.8 (98.8)	99.8 (99.4)	98.4 (99.5)	98.3 (99.1)	98.4 (98.7)	98.3 (98.4)
Mean *I*/σ(*I*)	16.84 (1.68)	10.18 (1.74)	10.39 (1.73)	10.24 (1.71)	11.46 (1.75)	11.46 (1.78)	11.34 (1.78)	11.37 (1.67)
*R*_meas_ ‡	0.061 (0.981)	0.103 (0.826)	0.101 (0.846)	0.102 (0.830)	0.096 (0.945)	0.095 (0.933)	0.096 (0.937)	0.097 (0.980)
CC_1/2_	0.999 (0.568)	0.997 (0.783)	0.998 (0.791)	0.998 (0.775)	0.998 (0.804)	0.996 (0.762)	0.996 (0.782)	0.998 (0.757)
Refinement statistics								
Resolution (Å)	38.40–1.38 (1.42–1.38)	38.55–1.70 (1.74–1.70)	38.55–1.70 (1.74–1.70)					
*R*_work_	0.140 (0.268)	0.194 (0.289)	0.190 (0.287)					
*R*_free_	0.170 (0.288)	0.239 (0.335)	0.216 (0.391)					
No. of atoms	1230	1208	1212					
Average atomic *B* factor (Å^2^)	16.6	23.5	23.7					
R.m.s. deviations								
Bond lengths (Å)	0.006	0.004	0.005					
Bond angles (°)	1.35	1.60	1.64					
PDB code	6qqh	6qqi	6qsa					

**Table:** 

	Light-100K D8	Light-100K D9	Light-100K D10	Light-100K D11	Light-100K D12	Light-100K D13	Light-100K D14	Light-100K D15
Data collection								
Temperature (K)	100							
Accumulated dose (MGy)	0.190	0.214	0.238	0.262	0.286	0.309	0.333	0.357
ESRF beamline	ID29							
Wavelength (Å)	0.976							
Space group	*P*4_3_2_1_2							
*a*, *b*, *c* (Å)	40.38, 40.38, 131.42	40.39, 40.39, 131.43	40.39, 40.39, 131.43	40.39, 40.39, 131.43	40.33, 40.33, 131.31	40.34, 40.34, 131.32	40.34, 40.34, 131.33	40.35, 40.35, 131.35
Resolution range† (Å)	38.61–1.71 (1.77–1.71)	38.61–1.72 (1.78–1.72)	38.61–1.71 (1.77–1.71)	38.56–1.70 (1.76–1.70)	38.56–1.71 (1.77–1.71)	38.56–1.71 (1.77–1.71)	38.56–1.71 (1.77–1.71)	38.57–1.71 (1.77–1.71)
Wilson *B* factor (Å^2^)	29.5	29.4	29.6	29.4	29.5	29.4	29.5	29.5
Unique reflections	12371 (1186)	12182 (1160)	12390 (1205)	12664 (1199)	12453 (1188)	12463 (1200)	12467 (1204)	12475 (1203)
Multiplicity	6.29 (6.44)	6.28 (6.39)	6.29 (6.44)	6.24 (6.46)	6.23 (6.38)	6.24 (6.40)	6.23 (6.40)	6.24 (6.45)
Completeness (%)	98.2 (97.5)	98.3 (99.1)	98.3 (99.3)	99.8 (99.2)	99.8 (98.3)	99.8 (99.3)	99.8 (99.3)	99.8 (99.3)
Mean *I*/σ(*I*)	11.37 (1.70)	11.60 (1.77)	11.18 (1.67)	10.06 (1.66)	10.27 (1.67)	10.23 (1.68)	10.12 (1.70)	10.06 (1.65)
*R*_meas_ ‡	0.096 (0.975)	0.095 (0.937)	0.098 (0.983)	0.103 (0.879)	0.101 (0.875)	0.102 (0.855)	0.103 (0.837)	0.104 (0.875)
CC_1/2_	0.997 (0.741)	0.998 (0.783)	0.998 (0.760)	0.997 (0.705)	0.997 (0.727)	0.996 (0.775)	0.997 (0.758)	0.997 (0.743)

**Table:** 

	Dark 293K	Light-293K D1	Light-293K D2	Light-293K D3	Light-293K D4
Data collection					
Temperature (K)	293				
Accumulated dose (MGy)	0.354	0.034	0.068	0.102	0.136
ESRF beamline	ID30A-3				
Wavelength (Å)	0.968				
Space group	*P*4_3_2_1_2				
*a*, *b*, *c* (Å)	40.886, 40.886, 132.691	41.45, 41.45, 133.53	41.45, 41.45, 133.53	41.45, 41.45, 133.53	41.45, 41.45, 133.53
Resolution range† (Å)	39.07–2.08 (2.15–2.08)	39.59–2.40 (2.50–2.40)	39.59–2.60 (2.70–2.60)	39.59–2.76 (2.86–2.76)	39.59–2.78 (2.88–2.78)
Wilson *B* factor (Å^2^)	43.1	52.0	59.6	63.0	67.7
Unique reflections	6924 (594)	4926 (558)	3897 (394)	3275 (278)	3217 (267)
Multiplicity	8.56 (8.73)	23.1 (23.5)	23.1 (22.0)	23.4 (21.1)	23.4 (21.1)
Completeness (%)	93.6 (87.4)	98.0 (99.1)	97.6 (99.0)	97.4 (88.8)	97.5 (86.4)
Mean *I*/σ(*I*)	10.72 (1.73)	17.64 (1.52)	19.14 (1.69)	19.69 (2.47)	17.12 (1.72)
*R*_meas_ ‡	0.145 (1.169)	0.177 (2.108)	0.160 (1.749)	0.154 (1.208)	0.184 (1.639)
CC_1/2_	0.999 (0.501)	0.999 (0.726)	0.999 (0.727)	0.999 (0.760)	0.999 (0.712)
Refinement statistics					
Resolution (Å)	24.21–2.08 (2.14–2.08)	39.59–2.40 (2.46–2.40)			
*R*_work_	0.247 (0.348)	0.239 (0.322)			
*R*_free_	0.292 (0.338)	0.286 (0.314)			
No. of atoms	956	1043			
Average atomic *B* factor (Å^2^)	38.7	46.9			
R.m.s. deviations					
Bond lengths (Å)	0.002	0.002			
Bond angles (°)	1.24	1.47			
PDB code	6qqj	6qqk			

† The resolution cutoff is based on CC_1/2_.

‡ *R*_meas_ = *R*_merge_ × [*N*/(*N* − 1)]^1/2^, where *N* is the data multiplicity.

**Table 5:** Global and specific radiation-damage dose constants

Protein	Cerulean		Lysozyme		*At*Phot2LOV2 photoadduct	
Temperature (K)	100	293	100	293	100	293
*D*_1/2_ (kGy)	8790	134	9190	105	32700	169
τ_Glob_ (kGy)	17600	308	18200	251	34300	389
τ_Spec_ (kGy)	843	105	1475, 10500†	198	22, 387†	49
Δ_G/S_	20.8	2.9	12.4	1.3	1590	8.0

† The specific damage curve for HEWL at 100 K is best fitted by two exponential decays, as observed previously (Carpentier *et al.*, 2010 ▸), which suggests the existence of an X-ray-induced repair mechanism. The same is likely to apply to the specific damage to the covalent bond in the *At*Phot2LOV2 photoadduct.
